# Intra-abdominal focal fat infarction of the omentum: diagnosis and percutaneous management

**DOI:** 10.1259/bjrcr.20150134

**Published:** 2015-07-03

**Authors:** V Chauhan, J A Stephenson, V Shah

**Affiliations:** Department of Radiology, Leicester Royal Infirmary, University Hospital of Leicester, Leicester, UK

## Abstract

We present a case of a 68-year-old female who presented with left iliac fossa pain and postprandial vomiting for 7 days. Initial CT scan revealed a large mass within the upper abdomen in close proximity to the pancreatic tail, with central fat density, marginal enhancement, a distended vessel coursing through the centre and hazy increased density of the fat outside of the structure. A diagnosis of intra-abdominal focal fat infarction (IFFI) was made and she was treated conservatively. Subsequently, she re-presented with further pain and elevated inflammatory markers. A repeat CT scan again demonstrated the well-defined mass, but now with the development of fluid attenuation within the centre and several pockets of air. Radiologically guided percutaneous drains were inserted into the area of liquefaction and subsequent microbiology analysis revealed mixed coliform bacilli; targeted antibiotics were administered. The collection resolved on subsequent imaging. Identification of radiological features of IFFI is very helpful in establishing a diagnosis and may negate the need for surgical intervention.

## Clinical presentation

A 68-year-old female presented to the surgical department with left iliac fossa pain and postprandial vomiting for 7 days. She reported the pain had been occurring for the past year but now had worsened. She had no change in bowel habit, haematemesis or haematochezia. She had a Whipple’s procedure 5 years previously, and 7 years previously had undergone a lumpectomy for breast malignancy. On examination, she was apyrexial and had left iliac fossa tenderness. Her white cell count was 12.8 × 10^9 ^l^–1^, with a neutrophil count of 8.9 × 10^9^ l^–1^ and a C-reactive protein (CRP) of 53 mg l^–1^.

## Image findings

**Figure 1.**
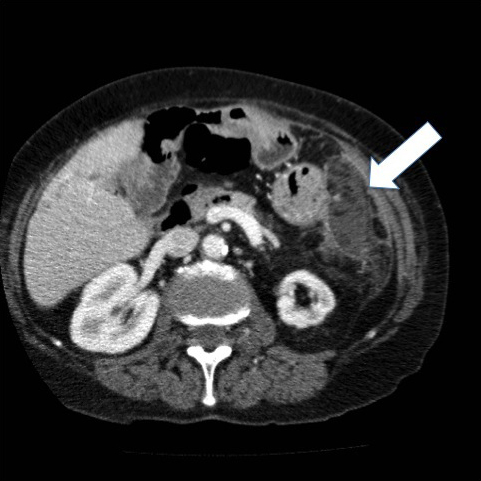
Axial portal venous phase contrast-enhanced CT: arrow shows left upper quadrant fat density mass with peripheral enhancement adjacent to the greater curve of the stomach.

**Figure 2.**
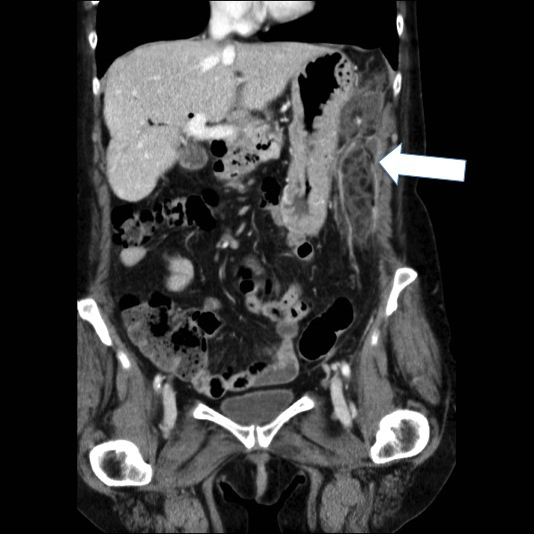
Coronal portal venous phase contrast-enhanced CT: arrow shows left upper quadrant fat density mass with peripheral enhancement adjacent to the greater curve of the stomach.

A CT scan of her abdomen and pelvis performed 4 weeks previously for follow-up of her pancreatic disease showed mild sigmoid diverticulosis. A repeat contrast-enhanced CT scan was performed to confirm suspicion of acute sigmoid diverticulitis. However, it revealed a large mass within the left upper quadrant, with central fat density, marginal enhancement, a distended vessel coursing through the centre and hazy increased density of the fat outside of the mass. Uncomplicated sigmoid diverticulosis was noted and unchanged (Figures 1 and 2).

CT scan confirmed a diagnosis of partial greater omental infarct, an entity within the broader diagnostic category of “intra-abdominal focal fat infarction (IFFI)”. A conservative management pathway was followed and she was discharged with analgesics 2 days later.

## Clinical presentation: re-admission

The patient re-presented 2 weeks later with further pain in the left iliac fossa and epigastrium and persistent postprandial vomiting, anorexia and weight loss. Her white cell count had climbed to 22.7 × 10^9^ l^–1^, with a neutrophil count of 20.04 × 10^9^ l^–1^ and a CRP of 210 mg l^–1^.

**Figure 3.**
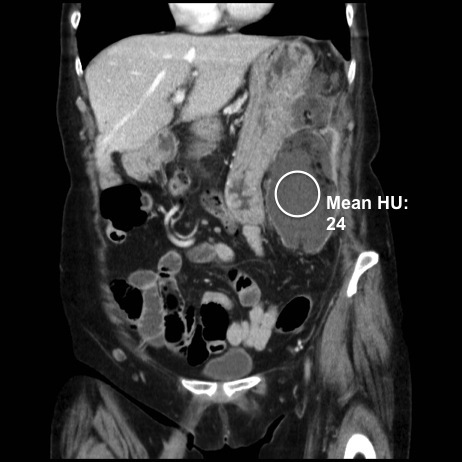
Coronal portal venous phase contrast-enhanced CT: circle represents region of interest measurement tool within left upper quadrant liquefied collection with foci of gas.

A repeat CT scan showed enlargement of the previously noted left upper quadrant mass, with new central fluid attenuation and several pockets of gas; no hollow viscus perforation could be identified and there was no free peritoneal gas (Figure 3).

## Differential diagnosis

Other causes for the left upper quadrant collections include acute/acute-on-chronic pancreatitis—with associated collection/pseudocyst, inflammatory carcinoma of the large bowel, acute diverticulitis with perforation and "contained" gastric perforation; however, these would not demonstrate a lesion of fat density.

The differentials for intra-abdominal fat density mass lesions with surrounding inflammatory change would include epiploic appendagitis (EA), omental infarction and mesenteric panniculitis.

## Treatment and follow-up

**Figure 4.**
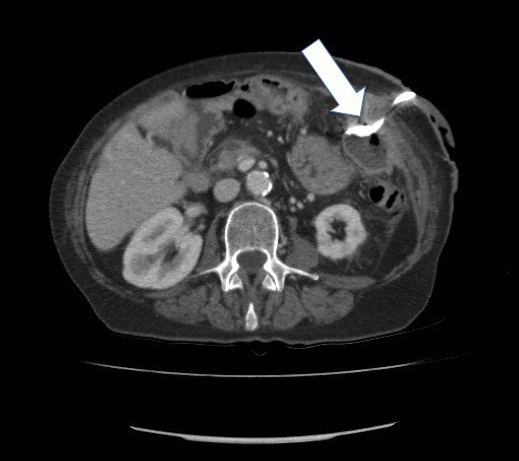
Axial portal venous phase contrast-enhanced CT: arrow shows decreased size of left upper quadrant collection with interval insertion of a pigtail drain.

It was surmised the area of fat liquefaction had become infected. As such, percutaneous drains were inserted under ultrasound guidance, and a repeat CT scan showed the collection had decreased in size (Figure 4).

**Figure 5.**
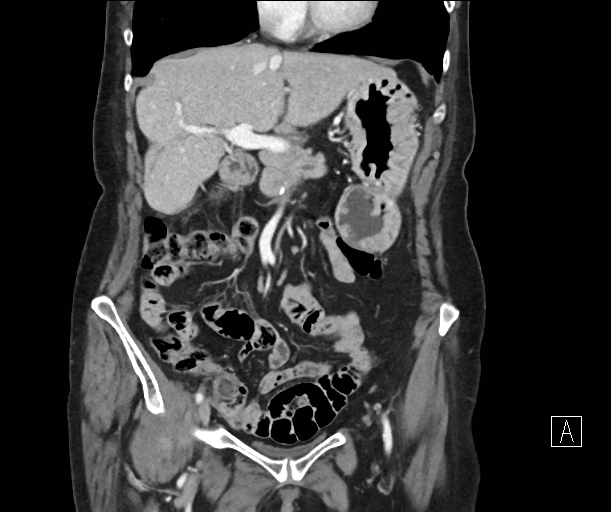
Coronal portal venous phase contrast-enhanced CT: complete resolution of the left upper quadrant collection.

Microbiological analysis of the drained material revealed mixed coliform bacilli and targeted antibiotic therapy was administered. An amylase level performed on the sample was negative (5 IU l^–1^), thus excluding leakage of pancreatic fluid. Symptoms improved with drainage of the liquefaction. She was discharged on day 11 after a marked reduction in drain contents and an improvement in her symptoms. A follow-up CT scan 10 months later showed complete resolution of the changes (Figure 5).

## Discussion

The term “intra-abdominal focal fat infarction” encompasses a range of conditions where infarction of fatty tissue is the underlying pathological process, including EA and segmental omental infarction, as well as rarer entities such as falciform ligament infarction.^1^

IFFI mimics more common intra-abdominal pathology and is frequently misdiagnosed.^2^ A radiological diagnosis based upon the specific features is helpful as IFFI can be managed conservatively with oral anti-inflammatory therapy^3^ and correct diagnosis negates further investigation and potential surgical treatment.^4^ Further imaging may be required when symptoms and signs deteriorate on a conservative management pathway, as complications such as fat necrosis, liquefaction and secondary abscess formation may be identified and prompt a change in management such as in this case. Laparoscopic resection of the inflamed areas may prevent recurrence and other complications.^3^

IFFI pathogenesis is torsion and vascular compromise of the fatty structure with engorgement of central vessels and inflammation secondary to ensuing ischaemia. Distinguishing features can be identified on ultrasound, CT scan and MRI.^2,4^ Ultrasonography usually reveals a non-compressible, well-defined, hyperechoic, intra-abdominal mass fixated to the organ of origin at the site of tenderness. Haemorrhage may be suggested by a hypoechoic central region. On CT scan, omental infarction manifests as a fatty density lesion with central and surrounding inflammatory change, closely apposed to the organ or structure of origin. Visceral peritoneal inflammation may be seen as a peripheral rim of hyperattenuation. An area of haemorrhagic change may also be seen as the central area of higher attenuation. MRI is rarely used given the usually acute presentation of IFFI. Typical signal changes include central hyperintensity on *T*_1_ weighted images, although slightly less intense than normal peritoneum with loss of signal on fat-suppressed *T*_2_ weighted images. Peripheral inflammatory changes are displayed as hypointense on *T*_1_ weighted images, hyperintense on *T*_2_ weighted images and enhancing on contrast enhanced fat-suppressed *T*_1_ weighted images.^5^

## Learning points

1. The term “intra-abdominal focal fat infarction” encompasses a range of conditions where infarction of fatty tissue is the underlying pathological process.
2. IFFI mimics more common intra-abdominal pathology and is frequently misdiagnosed. A radiological diagnosis based upon the specific features is helpful as IFFI can be managed conservatively with oral anti-inflammatory therapy and correct diagnosis negates further investigation and potential surgical treatment.
3. Further imaging may be required when symptoms and signs deteriorate with conservative management, as complications such as fat necrosis, liquefaction and secondary abscess formation may have resulted.
